# Development of a Dementia-Focused End-of-Life Planning Tool: The LEAD Guide (**L**ife-Planning in **E**arly **A**lzheimer’s and **D**ementia)

**DOI:** 10.1093/geroni/igz024

**Published:** 2019-08-02

**Authors:** Kara Dassel, Rebecca Utz, Katherine Supiano, Sara Bybee, Eli Iacob

**Affiliations:** 1 College of Nursing, University of Utah, Salt Lake City; 2 College of Social and Behavioral Sciences, University of Utah, Salt Lake City

**Keywords:** Advance care planning, Advance directive, Caregiving, Goals of care discussions, Palliative care

## Abstract

**Background and Objectives:**

To address the unique characteristics of Alzheimer’s disease and related dementias (ADRD) that complicate end-of-life (EOL), we created, refined, and validated a dementia-focused EOL planning instrument for use by healthy adults, those with early-stage dementia, family caregivers, and clinicians to document EOL care preferences and values within the current or future context of cognitive impairment.

**Research Design and Methods:**

A mixed-method design with four phases guided the development and refinement of the instrument: (1) focus groups with early-stage ADRD and family caregivers developed and confirmed the tool content and comprehensiveness; (2) evaluation by content experts verified its utility in clinical practice; (3) a sample of healthy older adults (*n* = 153) and adults with early-stage ADRD (*n* = 38) completed the tool, whose quantitative data were used to describe the psychometrics of the instrument; and (4) focus groups with healthy older adults, family caregivers, and adults with early-stage ADRD informed how the guide should be used by families and in clinical practice.

**Results:**

Qualitative data supported the utility and feasibility of a dementia-focused EOL planning tool; the six scales have high internal consistency (α = 0.66–0.89) and high test–rest reliability (*r* = .60–.90). On average, both participant groups reported relatively high concern for being a burden to their families, a greater preference for quality over length of life, a desire for collaborative decision-making process, limited interest in pursuing life-prolonging measures, and were mixed in their preference to control the timing of their death. Across disease progression, preferences for location of care changed, whereas preferences for prolonging life remained stable.

**Discussion and Implications:**

The LEAD Guide (Life-Planning in Early Alzheimer’s and Dementia) has the potential to facilitate discussion and documentation of EOL values and care preferences prior to loss of decisional capacity, and has utility for healthy adults, patients, families, providers, and researchers.

**Translational Significance**

The LEAD Guide can be used by healthy persons, those with early-stage ADRD, family caregivers, and health care professionals to inform EOL care decisions and ensure that they match the patient’s EOL values and preferences. Furthermore, the LEAD Guide can also be used in research settings to assess one’s EOL values and preferences and may be the basis of an intervention to support persons with dementia.

## Background and Objectives

There are currently around five million adults living with Alzheimer’s disease or related dementias (ADRD) in the United States. This number is expected to nearly triple to 13.9 million by 2060 (Matthews et al., 2019). Globally, the number of individuals with dementia is expected to rise from 4.7 million in 2015 to 132 million by the year 2050 (Prince et al., 2015). Alzheimer’s disease and related dementias (ADRD; e.g., vascular dementia, frontotemporal dementia, Lewy body dementia, etc.) precipitate decline in memory, thinking, and behavior, and worsen over time (Alzheimer’s Association, 2018). The later stages of dementias include difficulty initiating movement and walking, difficulty eating and swallowing, agitation, incontinence, and increased risk of pressure ulcers and infections, which invariably lead to death (Unroe & Meier, 2013).

With new technologies that extend the length of life, older adults are often cognitively impaired, due to a variety of medical conditions, at the end-of-life (EOL) and cannot make independent decisions about their own medical and EOL care (Carr & Khodyakov, 2007). An estimated 45%–70% of older adults facing EOL choices do not have the capacity to make their own treatment decisions as a result of various underlying diagnosis (IOM, 2014). Predictably, persons with ADRD due to the insidious progression of neurodegenerative diseases, medical decisions in the later stages are left to the patient’s health care agents or surrogates (IOM, 2014).

For individuals with ADRD, the lack of decisional capacity to make one’s own medical decisions at EOL impacts the treatments that they will or will not receive, and subsequently their physical and mental well-being at the end of life. Throughout this study, we use the terminology “ADRD” and “dementia.” For the purposes of this study, we define these terms as any irreversible progressive neurological condition or disorder that impairs cognitive functioning and subsequently decision-making capabilities. Additionally, the majority of family members of persons with advanced dementia state that comfort is their primary goal; however, very few of these patients are referred to hospice and many experience repetitive unproductive and distressing transitions between the hospital and nursing home (Unroe & Meier, 2013). However, advance care planning has been shown to increase rates of hospice utilization, reduce acute care interventions (e.g., resuscitation, artificial nutrition) and improve care outcomes in persons with dementia (e.g., reduced hospital and intensive care unit admissions, fewer visits to the emergency department, and more home versus hospital deaths) (Jennings et al., 2019; Mitchell et al. 2003).

The diminishing capacity associated with dementia increases the need for family members to make decisions on behalf of the person with ADRD. Frequently, families are unsure of how to make decisions, especially when they need to reflect the values and preferences of the person for whom they are caring. A recent study indicated that persons with dementia believed their family caregivers would know their values and EOL wishes and would advocate for them if they were unable to speak for themselves (Poole et al., 2018). While true for some, this same study reported that families felt distressed by the amount of “guess-work” they are faced with when having to make surrogate decisions on behalf of their relatives (p.7). Family members reported feeling ill-equipped to make health care decisions due to a lack of previous discussions regarding their relatives’ preferences for EOL care even though they had long-lasting and close relationships with the person with dementia (Poole et al., 2018).

In a systematic literature review, one-third of surrogate decision makers experienced emotional burden that lasted months or even years when tasked with making treatment decisions (Wendler & Rid, 2011). Surrogates suffered the most burden when the recommended treatment differed from the treatment the patient would have wanted. For family caregivers having to make unclear and unspecified life and death decisions on behalf of a relative with dementia, the impact can be long lasting and may involve stress, guilt, doubt, grief, and suicidal ideation (Wendler & Rid, 2011).

While advance directives—the legal document identifying a medical power of attorney and declaring one’s medical wishes—are designed to alleviate some of this burden, they are notably under-utilized. The Institute of Medicine (2014) found that more than 25% of all adults aged 75 years and older have given little or no thought to their EOL preferences and even fewer have had discussions with their family members or documented their wishes in writing. Once completed, advance directives are often not updated or are forgotten, placed in an inaccessible location, and not given to one’s doctor or included in the medical record (Carr & Luth, 2017; IOM, 2014). Even with access to completed advance directives, some physicians are hesitant to follow them due to perceived legal liability, and in some jurisdictions, they may not have legal standing (Carr & Luth, 2017; Howard et al., 2018). Advance directives are further constrained by legislatively-mandated language that limits their usefulness as a decision-making tool.

Advance care planning, defined as a provider and health team-facilitated process that enables individuals to plan their future health care, is intended to facilitate treatment goal clarity and the completion of advance directive documents. When conducted with clinical skill and accurate diagnostic and prognostic information, advance care planning provides direction to health care professionals and family caregivers for when patients are unable to self-advocate or communicate their own health care choices. Frequently, this type of planning occurs only in the later stages of chronic disease progression. With dementia, the discussion process begins too late, and cognitive impairment has progressed so that the scope of decisions and consequences of such decisions are not within the patient’s capacity to comprehend. These types of discussions are further complicated in the case of dementia, as individuals are planning for a “future unknown self” (Dixon, Karagiannidou, & Knapp, 2018). Some individuals with ADRD prefer to only give verbal wishes (Moss et al., 2018) because they do not want to limit their health care proxy’s decision-making options (Dixon et al., 2018).

Advance care planning discussions generally focus narrowly on health care decisions and do not include the values and preferences that encompass broader quality of life wishes such as location of care and location of death. The resultant advance directives are similarly constrained. While advance directives are useful in specific situations such as permanent coma or advanced cancer, or in the provision of interventional treatment such as artificial feeding, they do not address situations of progressive loss such as that experienced by persons with ADRD (Gaster, Larson, & Curtis, 2017).

Programs such as “The Conversation Project” (Institute for Health Care Improvement, 2019), “Me and My Wishes” (Towsley, Beck, Ellington, & Wong, 2018), and “Respecting Your Choices” (Prendergast, 2001) are examples of other patient and family-led efforts to discern and affirm EOL care preferences. These approaches emphasize communication between patients and families, as a way to clarify patient care preferences, yet do not incorporate cognitive-specific issues and do not have the flexibility to record probable care and treatment decisions that emerge with ADRD progression over time. Gaster and colleagues (2017) developed an advanced medical directive across dementia stages. However, its utility remains limited due to the focus solely on medical interventions and lack of validation.

### A New Advance Directive?

Given the limitations of current advance care planning, particularly its lack of specificity for ADRD patients, the clinical and research literature suggests a need for a new type of advance planning document as a national priority (Dixon et al., 2018). Advance care planning for persons with healthy adults, persons at risk of dementia, or those with early-stage ADRD ideally needs to be completed before dementia occurs and certainly before impairment progresses to the point that cognition and associated decision-making capabilities are impaired (Gaster, Larson, & Curtis, 2017; Thomas et al., 2018). In addition, an instrument specific for ADRD should be brief while addressing the changes in cognition and goals of care along the disease continuum (Gaster, Larson, & Curtis, 2017).

Carr (2011) has suggested that health care providers ask patients and families about their values, cultural practices and the history of their decision making—noting that a process that merely uses checkbox style options can be ineffective, inflexible and fail to anticipate future events and situations that emerge in dementia progression (IOM, 2014). In response, “combined directives” attempt to integrate legal advance directive documents with the patients’ values (President’s Council on Bioethics, 2005 as cited in Carr & Moorman, 2009, p.756).

Best practice recommendations suggest using a values-based approach instead of a treatment-based framework and repeating conversations over time as the illness progresses when working with cognitively impaired older adults (Thomas et al., 2018). Identifying a proxy decision maker and engaging in regularly scheduled goals-of-care discussions can help in clarifying treatment goals and exploring health care options as dementia progresses. Finally, Thomas et al. (2018) recommend using a structured tool or guide to ensure that discussions are captured and wishes documented to avoid ambiguity. Sharing these conversations with providers increases the likelihood that an individual’s wishes will be followed.

### Prior Research

In our earlier work (Dassel, Utz, Supiano, McGee, & Latimer, 2018), we used a sample of healthy older adults to examine EOL care preferences including preference for life-prolonging measures, willingness to engage in conversations about the timing of ones’ death, and preferences for location of death using three unique hypothetical death scenarios (pancreatic cancer, congestive heart failure, and Alzheimer’s disease). We found significant differences in the patients’ care preferences across these three diseases, most notably in their preferences related to location of death and use of life-sustaining treatment. These results suggested that EOL care preferences are particularly unique in situations involving dementia. These results also revealed that healthy persons are able to thoughtfully and critically engage in these types of EOL planning processes, imagining what EOL will be like under different disease scenarios.

Our previous research (Supiano, McGee, Dassel, & Utz, 2019) also used open-ended questions to classify underlying personal values that are distinct from one’s specific EOL preferences. Since values have been shown to predict EOL care preferences, we thought it essential to examine values in addition to preferences (Prendergast, 2001; Singer & Siegler, 1992; Winter, 2013). Our results demonstrated that reluctance to burden others was the most frequently voiced personal value across all conditions, followed by the value of quality (over length) of life.

The purpose of this study was to develop a valid and reliable tool that captures the EOL care preferences and values of individuals with ADRD. The goal was to create a tool that provides adequate information for a caregiver to assume decision-making responsibility when the individual with ADRD becomes incapacitated. Although this tool was developed with a dementia focus, this advance care planning tool has much broader potential utility for healthy adults and older adults who are concerned about their risk for ADRD, individuals with early ADRD, their family members, providers caring for such patients and their families, and researchers studying this population.

### Study Design

We used a mixed-method iterative design to create, modify and evaluate the suitability and value of a dementia-focused EOL planning tool. As given in Table 1, we conducted the current study in four phases, where each phase informed and guided the data collection and analysis of the next phase. We use the term “tool” to refer to the document that study participants and content reviewers received, we use the term “instrument” to refer to the psychometric properties of the tool, and we use the term “guide” to refer to the comprehensive and user-friendly format to be used for dissemination and possible future intervention. Review and approval of this study and all procedures were approved by the University of Utah Institutional Review Board.

**Table 1.** Study Phases, Objectives, and Method to Create and Validate The LEAD Guide

Phase	Objective	Methods and outcomes
1	To obtain feedback from potential users about usability and comprehensiveness of tool	• Used enhanced cognitive interviewing with two focus groups (persons with early-stage ADRD and current/former ADRD family caregivers)
		• Comments and suggestions directed modification of EOL planning tool
2	To obtain feedback regarding the tool’s utility and ease of use within clinical practice settings	• Distributed EOL planning tool and questionnaire to ten content experts in four disciplines and three clinical specialties
		• Comments and suggestions directed further modification of EOL planning tool
3	To evaluate the psychometric properties (validity and reliability) of the tool	• Distributed electronic version of EOL planning tool to a national sample of healthy older adults (*n* = 153) and persons with early-stage ADRD (*n* = 38). Participants completed tool twice over a 2-week period.
		• Statistical analyses provided benchmark values and internal consistency of each scale, as well as a measure of test–retest reliability of each scale
4	To obtain feedback on the tool’s utility, especially whether it contains sufficient information for a caregiver to provide accurate substituted judgement for someone with ADRD	• Conducted focus groups with three potential types of users (healthy older adults, persons with early-stage ADRD, current/former family ADRD caregivers)
		• Comments and suggestions will be incorporated into the creation of a comprehensive guide that includes detailed instructions, definition of terms, and a user-friendly format.

*Note*: ADRD = Alzheimer’s disease and related dementias; EOL = End-of-life.

To create a dementia-focused EOL planning tool, we used a previous survey instrument which demonstrated that people have different EOL values and preferences across varying hypothetical disease scenarios. It included three EOL *values* (concern about being a burden, quality vs quantity of life, autonomy vs shared decision making) and three *preferences* for specific EOL treatments (location of long-term care, life-prolonging measures, controlling the timing of death). Across the four study phases, we refined and validated these six dimensions of the tool with the goal of creating a useful instrument for researchers interested in measuring EOL values and preferences in healthy adults, persons at risk of ADRD, or persons diagnosed with ADRD and their caregivers and clinicians who will be the ones navigating EOL care decisions as decisional capacity is lost across the disease trajectory.

## Method and Results

### Phase 1

We conducted two focus groups using enhanced cognitive interviewing (Beatty & Willis, 2007; Krueger, 1998, 1998; Morgan, 1997; Willis, 2005) to obtain feedback from participants about the usability and comprehensiveness of the dementia-focused EOL planning tool. Cognitive interviewing is a method used for identifying and revising problems with survey questions by providing participants with a draft survey and obtaining verbal feedback about the survey responses (Beatty & Willis, 2007). The first group consisted of six individuals with early-stage ADRD and the second group consisted of 11 current or former dementia family caregivers. Participants were informed of the study aims and purpose of the focus groups prior to obtaining informed consent. Early-stage ADRD was self-identified by the patients and/or caregiver based on known diagnoses or conversations with a physician. Participants were on average 65.5 years old, mostly female (59%), non-Hispanic White (70.6%), married (82.4%), and well educated (41% had postgraduate education or a professional degree). Participants were recruited with assistance from the Center for Clinical and Translational Science at the University of Utah through a local memory clinic, the State Chapter of the Alzheimer’s Association, and local community-based aging programs, and conducted in the Center for Clinical and Translational Science offices.

Each focus group lasted 90 min. Participants completed the EOL planning tool and were asked to take specific notes regarding any questions or comments they wished to discuss. Facilitators with extensive research experience in focus groups (K. Supiano and S. Bybee) led each group in a discussion about the tool’s utility, acceptability, and language that was unclear or not applicable. The specific questions asked of participants were: (1) Please tell us about the wording of the document: (a) Were there any items that were not clear to you? (b) Were there any words that you did not understand? (c) Were there any questions that you felt did not apply to you? (d) Does the title of the document make sense?, (2) Do you understand the difference between the terms *values* and *preferences* used in the document? If no, what terms would help you better understand the distinction between the two?, (3) Do you feel comfortable answering “Please explain how confident you are about your end-of-life care preferences being carried out by your family members, health care proxy, and/or health care provider” knowing that it would be shared with your caregiver and/or doctor?, and (4) How does religion or spirituality factor into your values and preferences for end-of-life care? Researchers served as scribes for each focus group. Each session was also audiorecorded and transcribed.

Researchers reviewed all notes and transcriptions for shared comments and recommendations, distinguishing those made by caregivers and individuals with early-stage ADRD. Overall, both groups had similar feedback. They agreed that the EOL planning tool is important and has utility, particularly during the very early stages of dementia. Both groups commented that it serves as an impetus for challenging, yet essential, discussions. The most frequent suggestions we received included: (1) adding more detailed instructions about how and when to complete the tool; (2) adding an “uncertain at this time” option in preference questions; (3) removing questions about “confidence that one’s EOL decisions would be carried out” due to concern that it could precipitate paranoia in later-stage dementia; and (4) modifying the format to clarify and better capture how current preferences differ from future preferences. Comments and suggestions from focus group sessions were utilized to modify the EOL planning tool further.

### Phase 2

We distributed the revised tool to 10 content experts to obtain feedback regarding its utility, particularly within the clinical setting to be used for patients with early cognitive impairment and their family caregivers. Content experts included physicians, nurse practitioners, social workers, and care managers in geriatrics, neurology, family medicine, and palliative care. The content experts were provided with the tool and a survey asking them about individual items and wording. For example, “Does the individual item below measure the concept noted above in red?.” Response options included “Yes,” “No,” and “Maybe.” The concepts included: (a) documentation of end of life wishes, (b) concerns about being a burden, (c) attitudes about quality versus length of life, (d) attitudes about decision making, (e) preferences about location for long-term care, (f) preferences for life-prolonging measures, and (g) preferences for controlling the timing of their death. Additional open-ended questions included: (1) What would you add or remove from this planning guide? (2) What do you think about the length of the document? Is it too short or too long? (3) Would you review the completed planning guide with patients and families in an office visit? Describe how you might use this with your patients and families? (4) Would this planning guide be suitable to use in a Medicare annual visit? Why or why not? (5) Were there any terms used that patients may not understand? Please be specific and offer suggestions on how you discuss these concepts with your patients. (6) Do you have any additional questions, comments, or concerns as we refine this type of planning guide?

We tabulated and examined the content experts’ written answers, and where there was consensus (defined as an endorsement from a majority of reviewers) we followed their recommendations regarding about items to add or remove from the tool (e.g., removing questions regarding physician-assisted death due to the illegality of the option for persons with cognitive impairment), noting in particular any feedback received about when question wording did not match the intended concept (e.g., “quantity of life” vs “length of life), as well as adding clarifying language and definitions for some items (e.g., defining “physical” burden). The content experts also provided feedback on whether and how they might use the tool in their clinical practice—due to the length of the tool, the majority of content experts would provide it to their patients to complete at home and then schedule a separate visit to review and discuss care preferences.

This version had a Flesch Kincaid (an assessment of readability; Flesch, 1979) 11th-grade reading score. Grade level readability was determined using an online calculator: http://www.readabilityformulas.com/free-readability-formula-tests.php). Although this does not meet the average 8th-grade U.S. reading level (Davis & Wolf, 2004), it is more easily readable than the Utah State advance directive form, which has a 12th-grade reading level score. Due to the unavoidable use of multisyllable terminology such as “medical power of attorney,” or “do not resuscitate order,” we accepted this version for the next phase of development.

### Phase 3

This version of the EOL planning tool was electronically distributed to a total of 191 older adults (age 50+) for psychometric evaluation. Participants were recruited through two national research databases: ResearchMatch and TrialMatch. ResearchMatch is a national health research volunteer research registry supported by the U.S. National Institutes of Health as part of the Clinical Translational Science Award program (https://www.researchmatch.org). TrialMatch is a free matching service that connects individuals with Alzheimer’s, caregivers, and healthy volunteers to authorized clinical research studies (https://trialmatch.alz.org). Participants were instructed to complete the survey twice over the course of 2 weeks to assess reliability of the instrument. Data were collected and managed using REDCap electronic data capture tools (Harris et al., 2009).

The sample was split into two subsamples, representing older healthy adults (*n* = 153) and persons with early-stage ADRD (*n* = 38). Reflecting the profiles of the databases from which they were drawn, both subsamples were well-educated, with over half of each having a college degree or more, and were predominately non-Hispanic White (85% and 92%, respectively).

As given in Table 2, the individual items of the EOL planning tool were recoded and transformed into six scales representing the three EOL *value* constructs and the three EOL *preference* constructs identified by exploratory factor analysis done in previous research (Dassel et al., 2018) and confirmed as important by participants in Phase 1. To compute each scale, individual items were summed, divided by the total number of questions answered, then multiplied by the total number of questions. To handle item-missing data, we required at least 66% of the items to be completed. Thus, no missing data were imputed, and scaled scores were consistent across participants. Statistical analyses, using SPSS version 24 (IBM, 2016), were used to benchmark the measurement properties, to assess internal consistency, and to evaluate test–retest reliability of each scale included on the instrument. Identifying statistically significant differences across the two subsamples was not the primary goal and may not be realistic given the relatively small sample sizes, but we chose to split the sample into healthy and ADRD persons, so we could compare descriptive properties of each scale across the two subsamples as a way to explore EOL differences that may emerge across different populations using this tool or across the disease trajectory associated with ADRD.

**Table 2.** Individual Items and Construction of EOL Values and Preferences Scales

	Scales and individual items	Response options	Scale construction
**Values to Guide EOL Planning**	Concern About Being Burden		
	• I am concerned about being a financial burden to family or close friends	1 = strongly disagree 2 = disagree 3 = neither agree/disagree 4 = agree 5 = strongly agree	Sum of three items. Range 3–15, with higher numbers indicating higher concern for being a burden to family and friends.
	• I am concerned about being an emotional burden to my family or close friends.		
	• I am concerned about being a physical burden to my family or close friends. (Physical burden includes assistance bathing, toileting, transferring, or time spent providing care)		
	Importance of Quality Life (as opposed to length of life)		
	• Quality of life is more important than length of life.	1 = strongly disagree 2 = disagree 3 = neither agree/disagree 4 = agree 5 = strongly agree	Sum of four items. Range 4–20, with higher numbers indicating higher value for quality life (as opposed to long life).
	• Length of life is more important than quality of life. (reverse code)		
	• Given the choice, I would prefer to live a shorter but more satisfying life.		
	• I prefer to live as long as I can even if longer life is not of the highest quality. (reverse code)		
	Preference for Autonomous Decision Making		
	• In general, I prefer that end-of-life decisions be made by:	3 = me only, 2 = me with assistance and advice of family, my doctor, or both, 1 = my family alone, my doctor alone, or my family and doctor together (without my input)	Sum of five items. Range 5–15, with higher numbers indicating greater preference for autonomous decision making.
	• I prefer decisions related to location of ongoing care be made by:		
	• I prefer decisions related to location of death be made by:		
	• I prefer decisions related to life-prolonging measures be made by:		
	• I prefer decisions related to controlling when I die be made by:		
**Preferences for EOL Care**	Use of Life-Prolonging Measures		
	I would want to live as long as possible, • Even if I had to be on a life support or breathing machine.	2 = Yes 1 = Uncertain 0 = No	Sum of four items. Range 0–8, with higher numbers indicating greater preference for the use of life-prolonging measures.
	• Even if my brain had stopped working.		
	• Even if I had to be fed through a tube.		
	• Even if I were in severe pain. *Would your preference for life-prolonging measures change if you can no longer make your own decisions (yes, no)? If yes, repeat the 4 questions again with same responses*		
	Controlling the Timing of Death		
	• I would consider ending my own life by not eating or drinking.	2 = Yes 1 = Uncertain 0 = No	Sum of three items. Range 0–6, with higher numbers indicating greater preference for controlling the timing of death
	• I would consider independently ending my own life through self-directed means.		
	• I would consider taking a prescription medication to end my life, under the supervision of a physician (if legal in my state and if I were deemed competent) *Note: could not ask whether these preferences might change when one no longer has capacity to make decisions because these options are not legally or practically available at that point.*		
	Location of Care		
	If you were to require 24-hr care and supervision today, where is your preferred location to receive this care? (please select only one option) *Would your preference for the location of care change if you can no longer make your own decisions? (yes, no) If yes, repeat the question again with same responses*	In my home, in someone else’s home (please specify), in residential hospice (if available), in nursing home, in hospital, uncertain	Single item. Categorical measurement. No scale score created.

*Note*: EOL = End-of-life.

To assess internal consistency of the items used to measure each of the six EOL value and preference constructs, we calculated Cronbach alpha for each. As given in Table 3, the Cronbach alpha values range from 0.66 to 0.89. Current practice suggests that alpha values at or above 0.9 are considered to have *excellent* internal consistency; from 0.8 to 0.9 are considered *good*, 0.7 to 0.8 are *acceptable*, 0.6 to 0.7 are *questionable*, and below 0.5 are *unacceptable* (DeVellis, 2012; Kline, 2000). Except for the “preference for controlling when you die” among the early-stage ADRD group (α = 0.66), all of the other scales are in the excellent or good range, indicating high internal consistency across the items that are used to create each scale. Internal consistency is a measure of interitem correlation and indicates the degree to which a set of items measures a single latent construct. The construct representing one’s “location preference for ongoing care” was not included in these statistical analyses since it was measured with a single-item categorical response.

**Table 3.** Internal Consistency (as measured by Cronbach Alpha) of EOL Values and Preference Scales in Healthy Older Adult (*n* = 153) and Early-Stage ADRD (*n* = 38) Samples

	Healthy old age	Early-stage ADRD
Concern About Being Burden	0.87	0.89
Importance of Quality Life	0.84	0.84
Preference for Autonomous Decision Making	0.81	0.78
Use of Life-Prolonging Measures	0.75	0.83
Controlling the Timing of Death	0.81	0.66
Location of Care	--	--

*Note*: Cronbach Alpha reported in each cell. -- not calculated, single categorical item.

ADRD = Alzheimer’s disease and related dementias; EOL = End-of-life.

With internal consistency of the multi-item scales established, Table 4 presents descriptive statistics for the six *values* and *preferences* constructs, as reported by both the healthy older adult sample (*n* = 153) and the early-stage ADRD sample (*n* = 38). The first finding to note is that there were very little missing data. Four out of the six constructs were calculated on the full samples (no missing data); and in the other two scales, less than 3% of the samples was excluded due to missing data. This suggests that participants were able to navigate the instrument and did not feel it necessary to skip individual items. The second overall finding is the variation in scores found for each scale, with reported ranges closely matching the theoretical or possible range for each scale. For example, the full span of possible scores for “concern about being a burden” was captured (theoretical and observed range = 3–15). This finding suggests that the items included on the instrument plus the range of response options for each are specific enough to allow for and to capture individual variation in response. The descriptive results presented in Table 4 also provide benchmark values within the possible ranges for each scale. On average, respondents reported fairly high concern for being a burden to their families (mean = 11.13 and 11.49 for the older healthy and ADRD subsamples, out of a 3–15 range), a greater preference for quality over length of life (mean = 14.78 and 14.84, out of a 4–20 range), desire for a collaborative decision-making process (mean = 10.66 and 10.94, out of a 5–15 range), limited interest in pursuing life-prolonging measures (mean = 0.76 and 0.94, out of a 0–8 range), and mixed preferences to personally control the timing of their death (3.01 and 3.32, out of a 0–6 range). Preference for location of care is a single-item categorical variable and therefore cannot be interpreted on a continuum of responses as the other five scales can. However, this variable revealed that about half of participants want to receive long-term care in their own home. The next most preferred location was a residential hospice, with about one-quarter of respondents selecting that option. About one in ten were uncertain about where they prefer to receive ongoing care.

**Table 4.** Benchmark Values of EOL Values and Preference Scales in Healthy Older Adult (*n* = 153) and Early-Stage ADRD (*n* = 38) Samples

	Mean or %		Median	Mode	*SD*	Min	Max	*n*
Concern About Being a Burden	11.13		12	15	3.25	3	15	153
	11.49		12	15	3.64	3	15	38
Importance of Quality Life	14.78		15	18	2.96	5	18	153
	14.84		16	18	3.50	4	18	38
Preference for Autonomous Decision Making	10.94		10	1	1.68	6	15	148
	10.66		10	10	1.94	6	15	37
Use of Life-Prolonging Measures	0.94		0	0	1.58	0	7	153
	0.76		0	0	1.57	0	7	38
Controlling the Timing of Death	3.01		3	0	2.21	0	6	148
	3.32		3.5	2	2.08	0	6	38
Location of Care	*n* = 153	*n* = 38						
In my home	54.2%	50.0%						
In someone else’s home	1.3%	5.3%						
In residential hospice	26.8%	21.1%						
In nursing home	5.9%	10.5%						
In hospital	1.3%	0.0%						
Uncertain	10.5%	13.2%						

*Note*: Independent samples *t* tests found no statistically significant differences between the healthy older adult sample (*n* = 153) and the early-stage ADRD sample (*n* = 38).

ADRD = Alzheimer’s disease and related dementias; EOL = End-of-life.

As stated at the bottom of Table 4, independent samples *t* tests revealed that there were no statistically significant mean differences across the healthy and ADRD subsamples. As stated previously, quantitative comparison of the two subsamples is not the priority given the relatively small and unbalanced sample sizes. In general, however, we see very little, if any, statistical or substantive differences across the healthy old age and ADRD subsamples on the measurement properties of the EOL value and preferences scales, thus supporting the broader application of the tool in both healthy and ADRD populations.

As conceptualized, EOL *value*s are enduring and should remain fairly unchanged over the disease trajectory. Conversely, *preferences* for specific EOL care may potentially shift across disease trajectories. Given these conceptualizations, the instrument explicitly asked respondents whether they thought their *preference* might change once they lost decisional capacity. Only 15.9% of the healthy older adults and 10% of the early-stage ADRD samples reported that they thought their preference for using life-prolonging measures would change over the course of disease. Among those who did express an anticipated change, they reported being more willing to accept life-prolonging measures after they lost their decisional capacity. Regarding changes in where care was received, a much larger proportion of respondents suggested that their preferences would change as their dementia progressed. For example, among the healthy older adults, more than 40% said their preferred location of care would change, and most of the change is accounted for whether they want to be in their home. During early stages, 54% wanted to receive their care in the home, but during later stages of the illness when they can no longer make their own decisions, less than 10% preferred to be in their own home, 50% preferred to be in a residential hospice, and 11% in a nursing home. A very similar pattern emerged among the ADRD subsample, with 37% saying their preferred location for care would change when they were no longer able to make decisions regarding their care. Like the healthy older group, they too were less likely to prefer receiving care in their home in later-stage dementia (less than 7% of the sample) and expressed greater uncertainty (36% of the sample) about where care should be provided during the later stages of the disease.

Finally, Table 5 shows test–retest reliability of each EOL scale. Each respondent was asked to complete the instrument twice within a 2-week period. The instrument at Time 2 was precisely the same as the instrument at Time 1. Approximately 98% of the healthy older adult sample (*n* = 150 out of 153) and 74% of the early-stage ADRD sample (*n* = 28 out of 38) completed the instrument at both time points, with an average of about 15 days between the two completions (mean = 15.7 days, *SD* = 5.5 for the healthy older adult subsample; mean = 15.1 days, *SD* = 1.8 for the ADRD subsample). The test–retest reliability coefficients, as measured by Pearson correlation *r*, ranged from .60 to .90. All are statistically significant at *p* < .01. These results suggest that the instrument has a high level of reliability, meaning that it captures similar response when administered repeatedly to the same person. The lower response rate among the ADRD subsample may suggest challenges associated with memory and task completion that are associated with ADRD, and should be considered when thinking about how to most effectively use this tool over time with an ADRD population.

**Table 5.** Test–retest Reliability (as measured by Pearson Correlation) of EOL Values and Preference Scale in Healthy Older Adult and Early-Stage ADRD Subsamples

	Healthy old age	Early-stage ADRD
Concern About Being Burden	0.67**	0.60**
Importance of Quality Life	0.81**	0.78**
Preference for Autonomous Decision Making	0.75**	0.72**
Use of Life-Prolonging Measures	0.79**	0.90**
Controlling the Timing of Death	0.81**	0.77**
Location of Care	--	--

*Note*: Each scale was measured at two time points, 2 weeks apart. Pearson Correlation reported in each cell, comparing time 1 and time 2 measures. -- not calculated, single categorical item. **Correlation is statistically significant at *p* < .01 (two-tailed).

ADRD = Alzheimer’s disease and related dementias; EOL = End-of-life.

### Phase 4

Three final focus groups were conducted to gather insight and feedback on how the instrument could be further developed for use by consumers as a guide that encourages and facilitates EOL planning discussions that capture the dynamics and challenges of ADRD over time. Specific questions asked of participants were: (1) How can you foresee yourself using this document to help inform your care? (2) Would you want to share this document with your doctor? (3) Did filling out this document bring up any issues you had not previously thought about? If so, which issues? (4) Do you think completing this document will help you to have a conversation with your loved one about these topics? Why or why not?

The final focus groups consisted of one group of 14 participants who were current or former ADRD caregivers, another group of 10 healthy adults age 50+, and a third group of 6 adults with a diagnosis of early ADRD. Participants were on average 60.5 years of age and 67% female. The largest percentage of participants reported their race/ethnicity as non-Hispanic (97%) and White (60%). Fifty percent of the participants had some college or were college graduates, with 36.7% having postgraduate education. None had participated in earlier phases of this study. They were recruited using the same procedures as those in the Phase 1 focus groups. Focus groups lasted 90 min and were audio recorded. Recordings and notes from each session were evaluated both within and between the three groups to identify shared comments and recommendations.

Feedback received across all three groups focused on potential scenarios in which the EOL planning tool could be useful. For example: (1) it could be used to help neutralize conflict between family members especially in acute medical situations where highly emotional decisions need to be made quickly; (2) it has potential to empower persons with early ADRD to make their preferences known across the disease course, and (3) it could be used as a conversation starter to help guide family and health care providers through advance care planning specific to ADRD. Limitations identified included the lack of legal status associated with the completed tool and not adequately addressing the nuances of cultural variability. Overall, the participants found great value in this type of document and suggested that it makes a strong foundation for advance care planning prior to or within the dementia context, and would complement the existing legal documents that are associated with EOL care.

## Discussion and Implications

The primary purpose of this project was to create, refine, and validate a dementia-focused EOL planning tool that could be used by across a broad spectrum of users ranging from healthy adults and older adults to those diagnosed with early memory loss as well as family caregivers of persons with early memory impairment, providers caring for such patients and their families, and for researchers studying this population. Using a multimethod iterative process of instrument development, we modified an existing EOL instrument developed in our earlier research and then reviewed it with multiple stakeholders, incorporating their qualitative feedback into the design of the tool and using quantitative analyses to evaluate the psychometric properties of the instrument itself in both healthy older adults and persons with early ADRD. Our work to date has yielded an EOL planning tool that is specific to current or potential future context of ADRD, and that is valid, reliable, acceptable, and has multiple potential uses within families, clinical settings, or research applications.

A second goal of this study was to discern if the EOL preferences of persons with early memory loss or those anticipating memory loss might change with the progression of dementia. Our initial research indicated that while persons’ values are enduring and stable over time, their specific preferences for EOL care vary by disease (Supiano et al., 2019). Overall, medical treatment choices were stable for most of the sample—persons’ choices were unchanged by stage of disease. However, a higher number of respondents suggested that their preferred location of services and use of family versus professional supports did change by disease stage. During later stages of cognitive impairment, they were more uncertain of their preferred location of care and less likely to prefer in-home care with a greater preference for nursing home or residential hospice than they were during the early stages of dementia. Although using hypothetical future scenarios to capture potential change is not as accurate as measuring a construct with repeated measures (Gundersen, 2003), in the case of ADRD where cognitive decline is progressive and not curable, it is not feasible to measure preferences in a repeated measures framework when cognitive impairment is severe. Thus, having a person with early-stage ADRD think about and anticipate their preferences and potential changes in those preferences is an important and essential component to advance care planning within a dementia-focused context.

Our EOL planning tool expands upon existing care planning tools in several ways. First, the instrument is comprehensive, yet concise. It includes questions about multiple domains including the status of legal EOL documentation, *values* that may guide EOL care decisions, and *preferences* for specific types of EOL care. Within each of these three domains, the tool includes closed-ended questions to document one’s overall values and preferences, as well as open-ended questions to supplement the general views with more specific and personalized details. The opportunity to provide both breadth and depth across multiple domains is intended to facilitate conversations between families and providers, hopefully resulting in greater confidence and less stress among the surrogate decision makers for ADRD persons (Poole et al., 2018). Second, to our knowledge, this is the first dementia-focused EOL planning instrument developed using established instrument development procedures to determine psychometric validity and reliability. The current results demonstrated good internal consistency and test–retest reliability, allowing the tool to be used by researchers and clinicians who want to measure EOL values and preferences in healthy aging and ADRD populations.

Current results, especially those from the qualitative focus groups and content-expert reviews, suggest high need and utility for a dementia-focused EOL tool such as the one developed here. Nevertheless, this study is not without limitations. The current study followed rigorous instrument development procedures, yet the sample size for persons with early-stage ADRD was small, thus limiting the generalizability of the quantitative results from that subsample. However, the small sample appeared to be similar in most regards to the healthy older adult sample, and all groups expressed similar potential uses and recommendations for this type of EOL planning tool.

Similarly, although we achieved diversity within our focus groups, our quantitative sample was predominately comprised of White, female, well-educated adults. The highest prevalence of ADRD in the United States occurs in non-Hispanic White females, which is reflected in our sample, however, per-capita Black and Hispanic Americans are significantly more likely (two times and one and a half times, respectively) to have ADRD than non-Hispanic Whites (Alzheimer’s Association, 2019; Matthews et al., 2019). Also, since White adults are more likely to complete advance directives or EOL planning compared to persons in minority groups (Portanova, Ailshire, Perez, Rahman, & Enguidanos, 2017), further investigation of the effects of cultural differences in the utility of this guide is needed. Lastly, our EOL planning instrument is intentionally generic and is intended to serve as a foundation of one’s values and preferences about EOL care. As a result, it does not adequately address the complexity of family dynamics, cultural nuances, or individual circumstances that may influence an individual’s EOL planning. Future research studies could utilize the instrument to explore how EOL care planning might change across the ADRD disease trajectory and between persons of diverse and unique circumstances.

Based on the results of the current study, particularly the qualitative feedback we received from healthy older adults, persons with early ADRD, their caregivers, and clinicians who work with these populations, we intend to transform the EOL planning instrument into a user-friendly guide. This includes adding instructions with suggested tips and guidelines for each type of user (i.e., healthy older adults, those at risk of ADRD, and those diagnosed with early-stage ADRD) in a variety of settings (i.e., completed alone, in discussion with family, in discussion in a medical appointment with one’s provider). The guide will also include suggestions for addressing the emotional nature of the discussion and a summary of definitions and terms used in the tool. Graphics and text formatting will make it more user-friendly, and allow it to become the “LEAD Guide” (Life-planning in Early Alzheimer’s and Dementia). This type of guide is not only useful for the patients and families coping with the changes associated with ADRD but also has important research applications. Transformed into a comprehensive guide, it is the basis of interventions for individuals and families who have received or may in the future receive a diagnosis of ADRD.

In conclusion, we have followed a rigorous instrument development process to create and validate a dementia-focused EOL planning tool that has the potential to be used with families and in clinical settings to supplement and clarify advance directives. It may also prove useful as a valid and reliable tool to conduct research across a spectrum of cognitive abilities in relation to EOL planning. As organizations such as the Alzheimer’s Association and the U.S. Department of Health and Human Services increase their advocacy for early diagnosis of dementia, the LEAD Guide will serve as an ideal resource to facilitate both conversations and research about the unique EOL planning associated with dementia.

## Funding

This work was supported by the University of Utah College of Nursing Dick and Timmy Burton Research Fund; and the National Center for Advancing Translational Science at the National Institutes of Health, Center for Clinical and Translational Sciences Grant Support (grant numbers 8UL1TR000105, UL1RR025764).

## Conflict of Interest

None reported.

## References

[CIT0001] Alzheimer’s Association (2018) What is Alzheimer’s? Retrieved from https://www.alz.org/alzheimers_disease_what_is_alzheimers.asp

[CIT0002] Alzheimer’s Association (2019). 2019 Alzheimer’s disease facts and figures. Alzheimer’s & Dementia, 15, 321–387. doi:10.1016/j.alz.2019.01.010 21414557

[CIT0003] Beatty P. C., & Willis G. B (2007). The practice of cognitive interviewing. Public Opinion Quarterly, 71, 287–311. doi:10.1093/poq/nfm006

[CIT0004] Carr D (2011). Racial differences in end-of-life planning: Why don’t Blacks and Latinos prepare for the inevitable? Omega, 63, 1–20. doi:10.2190/OM.63.1.a 21748919

[CIT0005] Carr D., & Khodyakov D (2007). End-of-life health care planning among young-old adults: An assessment of psychosocial influences. The Journals of Gerontology, Series B: Psychological Sciences and Social Sciences, 62, S135–S141. doi:10.1093/geronb/62.2.s135 17379683

[CIT0006] Carr D., & Luth E. A (2017). Advance care planning: Contemporary issues and future directions. Innovation in Aging, 1, igx012. doi:10.1093/geroni/igx012 30480109 PMC6177019

[CIT0007] Carr D., & Moorman S. M (2009). End-of-life treatment preferences among older adults: An assessment of psychosocial influences. Sociological Forum (Randolph, N.J.), 24, 754–778. doi:10.1111/j.1573-7861.2009.01135.x PMC2971558 21057589

[CIT0008] Dassel K. B., Utz R., Supiano K., McGee N., & Latimer S (2018). The influence of hypothetical death scenarios on multidimensional end-of-life care preferences. The American Journal of Hospice & Palliative Care, 35, 52–59. doi:10.1177/1049909116680990 28273753

[CIT0009] Davis T. C., & Wolf M. S (2004). Health literacy: Implications for family medicine. Family Medicine, 36, 595–598. 15343422

[CIT0010] DeVellis R. F (2012). Scale development: Theory and applications (pp. 109–110). Los Angeles, CA: Sage.

[CIT0011] Dixon J., Karagiannidou M., & Knapp M (2018). The effectiveness of advance care planning in improving end-of-life outcomes for people with dementia and their carers: A systematic review and critical discussion. Journal of Pain and Symptom Management, 55, 132–150.e1. doi:10.1016/j.jpainsymman.2017.04.009 28827062

[CIT0012] Flesch R. F (1979). How to write plain English. New York, NY: Harper & Row.

[CIT0013] Gaster B., Larson E. B., & Curtis J. R (2017). Advance directives for dementia: Meeting a unique challenge. JAMA, 318, 2175–2176. doi:10.1001/jama.2017.16473 29114779

[CIT0014] Gundersen L (2003). Dispositional theories of knowledge. London, UK: Routledge.

[CIT0015] Harris P. A., Taylor R., Thielke R., Payne J., Gonzalez N., & Conde J. G (2009). Research electronic data capture (redcap)–a metadata-driven methodology and workflow process for providing translational research informatics support. Journal of Biomedical Informatics, 42, 377–381. doi:10.1016/j.jbi.2008.08.010 18929686 PMC2700030

[CIT0016] Howard M., Day A. G., Bernard C., Tan A., You J., Klein D., & Heyland D. K (2018). Development and psychometric properties of a survey to assess barriers to implementing advance care planning in primary care. Journal of Pain and Symptom Management, 55, 12–21. doi:10.1016/j.jpainsymman.2017.08.003 28803079

[CIT0017] IBM Corp (2016). IBM SPSS statistics for windows, version 24.0. Armonk, NY: IBM Corp.

[CIT0018] Jennings L. A., Turner M., Keebler C., Burton C. H., Romero T., Wenger N. S., & Reuben D. B (2019). The effect of a comprehensive dementia care management program on end-of-life care. Journal of the American Geriatrics Society, 67, 443–448. doi:10.1111/jgs.15769 30675898 PMC9859712

[CIT0019] Institute for Healthcare Improvement (2019). The conversation project: Your conversation starter kit Retrieved from https://theconversationproject.org/wp-content/uploads/2017/02/ConversationProject-StarterKit-Alzheimers-English.pdf

[CIT0020] Institute of Medicine (IOM) (2014). Dying in America: Improving quality and honoring individual preferences near the end of life. Washington, DC: The National Academies. 25927121

[CIT0021] Kline P (2000). The handbook of psychological testing (2nd ed.). London: Routledge pp. 7–8.

[CIT0022] Krueger R. A (1998). Moderating focus groups. In Morgan D. L. & Krueger R. A. (Eds.), The focus group kit (Vol. 4). Thousand Oaks, CA: Sage Publications.

[CIT0024] Matthews K. A., Xu W., Gaglioti A. H., Holt J. B., Croft J. B., Mack D., & McGuire L. C (2019). Racial and ethnic estimates of Alzheimer’s disease and related dementias in the United States (2015–2060) in adults aged ≥65 years. Alzheimer’s & Dementia, 15, 17–24. doi:10.1016/j.jalz.2018.06.3063 PMC6333531 30243772

[CIT0025] Mitchell S. L., Teno J. M., Roy J., Kabumoto G., & Mor V (2003). Clinical and organizational factors associated with feeding tube use among nursing home residents with advanced cognitive impairment. JAMA, 290, 73–80. doi:10.1001/jama.290.1.73 12837714

[CIT0026] Morgan D. L (1997). Focus groups as qualitative research. Thousand Oaks, CA: Sage Publications.

[CIT0027] Moss K. O., Deutsch N. L., Hollen P. J., Rovnyak V. G., Williams I. C., & Rose K. M (2018). End-of-life plans for African American older adults with dementia. The American Journal of Hospice & Palliative Care, 35, 1314–1322. doi:10.1177/1049909118761094 29540073 PMC6026577

[CIT0028] Poole M., Bamford C., McLellan E., Lee R. P., Exley C., Hughes J. C.,…Robinson L (2018). End-of-life care: A qualitative study comparing the views of people with dementia and family carers. Palliative Medicine, 32, 631–642. doi:10.1177/0269216317736033 29020864

[CIT0029] Portanova J., Ailshire J., Perez C., Rahman A., & Enguidanos S (2017). Ethnic differences in advance directive completion and care preferences: What has changed in a decade? Journal of the American Geriatrics Society, 65, 1352–1357. doi:10.1111/jgs.14800 28276051 PMC5893138

[CIT0030] Prendergast T. J (2001). Advance care planning: Pitfalls, progress, promise. Critical Care Medicine, 29, N34–N39. 11228571 10.1097/00003246-200102001-00007

[CIT0031] President’s Council on Bioethics (2005). Taking care: Ethical caregiving in our aging society. Washington, DC: Government Printing Office.

[CIT0032] Prince M., Wimo A., Guerchet M., Ali G. C., Wu Y. T., & Prina M (2015). World Alzheimer’s report 2015: The global impact of dementia: An analysis of prevalence, incidence, cost, and trends. London, England: Alzheimer’s Disease International.

[CIT0033] Singer P. A., & Siegler M (1992). Advancing the cause of advance directives. Archives of Internal Medicine, 152, 22–24. 1728923

[CIT0034] Supiano K. P., McGee N., Dassel K. B., & Utz R (2019). A comparison of the influence of anticipated death trajectory and personal values on end-of-life care preferences: A qualitative analysis. Clinical Gerontologist, 42, 247–258. doi:10.1080/07317115.2017.1365796 28990872

[CIT0035] Thomas J. D., Sanchez-Reilly S., Bernacki R., O’Neill L., Morrison L. J., Kapo J.,…Carey E. C (2018). Advance care planning in cognitively impaired older adults. Journal of the American Geriatrics Society, 66, 1469–1474. doi:10.1111/jgs.15471 30277566

[CIT0036] Towsley G. L., Beck S. L., Ellington L., & Wong B (2018). Me & my wishes: Lessons learned from prototyping resident centered videos about care preferences. Journal of Applied Gerontology, 37, 1037–1049. doi:10.1177/0733464816657473 27384047

[CIT0037] Unroe K. T., & Meier D. E (2013). Quality of hospice care for individuals with dementia. Journal of the American Geriatrics Society, 61, 1212–1214. doi:10.1111/jgs.12318 23855849

[CIT0038] Wendler D., & Rid A (2011). Systematic review: The effect on surrogates of making treatment decisions for others. Annals of Internal Medicine, 154, 336–346. doi:10.7326/0003-4819-154-5-201103010-00008. Retrieved from http://annals.org/aim/fullarticle/746856/systematic-review-effect-surrogates-making-treatment-decisions-others 21357911 10.7326/0003-4819-154-5-201103010-00008

[CIT0039] Willis G. B (2005). Cognitive interviewing. Thousand Oaks, CA: Sage Publications.

[CIT0040] Winter L (2013). Patient values and preferences for end-of-life treatments: Are values better predictors than a living will? Journal of Palliative Medicine, 16, 362–368. doi:10.1089/jpm.2012.0303 23442042

